# Conformation of HLA-E/peptide complex guides interaction with two novel HLA-E receptors: Stabilin 1 and 2

**DOI:** 10.1371/journal.pone.0334543

**Published:** 2025-10-13

**Authors:** Tom A. W. Schoufour, Linda Voogd, Kees L.M.C. Franken, Tom H.M. Ottenhoff, Ruud H.M. Wijdeven, Simone A. Joosten

**Affiliations:** 1 Department of Cell and Chemical Biology, Oncode Institute, Leiden University Medical Center, Leiden, The Netherlands; 2 Leiden University Center for Infectious Diseases, Leiden University Medical Center, Leiden, The Netherlands; 3 Department of Functional Genomics, Center for Neurogenomics and Cognitive Research, Vrije Universiteit Medical Center, Amsterdam, The Netherlands; University of Arizona, College of Medicine-Phoenix, UNITED STATES OF AMERICA

## Abstract

Human leukocyte antigen E (HLA-E) communicates cellular health to natural killer (NK) cells through presentation of peptides derived from the leader sequence of classical major histocompatibility complex class I (MHC-I), inhibiting NK cell activation and lysis of healthy cells. Besides this canonical role, HLA-E can also present peptides from pathogens such as *Mycobacterium tuberculosis* (Mtb) to T cells and can inhibit phagocytosis by engaging with LILRB1/2. To identify additional HLA-E binding surface molecules, we utilized a CRISPR/Cas9 activation screen with HLA-E tetramers, which identified Stabilin (STAB)1 and STAB2 as novel interactors. This interaction depended on the nature of the peptide/HLA-E complex, whereby high affinity peptides did not permit the interaction while low affinity peptides did. Functionally, expression of STAB1 or STAB2 on THP-1 monocytes increased phagocytic uptake of HLA-E coated microbeads. These results provide the first evidence of an interaction between Stabilin receptors and specific HLA-E conformations.

## Introduction

Human leukocyte antigen E (HLA-E) is a conserved non-classical major histocompatibility complex class I (MHC-I) molecule with an essential role in both innate and adaptive immunity [1–3]. While the allelic variation of classical HLA-A, -B -C molecules across the human population is large, HLA-E molecules have very limited diversity as only two allelic forms are functionally expressed among the human population [4,5]. These two alleles, HLA-E*01:01 and HLA-E*01:03, differ by one single amino acid located outside the peptide binding groove and their peptide binding repertoire has thus far been overlapping although not fully identical [6,7]. The monomorphic property of HLA-E is essential for its conventional role; to report cellular health to NK cells. In homeostasis, HLA-E presents nonameric self-peptides (VL9) originating from the leader sequences of classical MHC-I molecules [8,9] to CD94/NKG2A receptors, resulting in transduction of an inhibitory signal to NK cells that prevents lysis of the presenting cell [10,11]. This interaction provides NK cells with “missing self” recognition; the capability to clear cells with insufficient MHC-I expression. The significance of this mechanism is illustrated by *cytomegalovirus* (CMV), which evolved to evade the immune system by using VL9-like peptides derived from its viral protein UL40. While CMV downregulates classical MHC-I to prevent recognition by the immune system, it presents UL40-derived peptides on HLA-E to prevent NK cell-mediated lysis, which ensures survival of CMV-infected cells [12,13]. Besides the canonical role of NK cell inhibition, HLA-E can act as a phagocytic inhibitor for myeloid cells via interaction with LILRB1 [14]. This interaction is independent of the presented peptide, since LILRB1 binds to the invariant β_2_ microglobulin (β_2_M) domain, its interaction with HLA-E is likely similar to the one with classical MHC-I [15,16]. Additionally, HLA-E can present pathogen or tumor-derived peptides to (CD8^+^) T cells that recognize the peptide/HLA-E complex via their T-cell receptor (TCR) [17]. TCRs have been identified that recognize peptides from intracellular bacteria and viruses specifically presented on HLA-E [2]. This concept has mostly been studied in the context of *Mycobacterium tuberculosis* (Mtb), and HLA-E/Mtb restricted CD8^+^ T cells seem to be unorthodox as these T cells can exert both regulatory, mycobacterium growth inhibitory and cytotoxic responses [18]. While this HLA-E/Mtb associated TCR repertoire is diverse, these interactions are highly variable and do not seem to induce strong clonal expansion [19].

The interaction with CD94/NKG2A is partly dependent on the conformation of the HLA-E/peptide complex, which is governed by the presented peptide [20]. The peptide binding groove (PBG) of HLA-E consists of two α-helices (α1 and α2) and a β-sheet floor. Peptide residues interact non-covalently with these α-helices and fit into one of the six pockets (A-F) in the β-sheet floor [21]. The canonical VL9 peptide conformation stabilizes the overall HLA-E/peptide complex, which results in a homogeneous and rigid HLA-E/peptide complex [22]. The high-affinity *Mtb*-derived peptide Mtb44 also induces this canonical conformation, forming a homogeneous and stable complex with HLA-E through compensatory interactions between the peptide residues and the PBG [21,22]. In contrast, the low-affinity HIV-derived peptide RL9HIV induces a structurally different non-canonical conformation of the HLA-E/peptide complex that is less stable and heterogeneous [21,22]. Other low-affinity *Mtb* peptides form similar heterogeneous HLA-E/peptide complex conformations and there is a strong positive correlation between peptide affinity and structural stability of the HLA-E/peptide complex. This low-affinity Mtb peptide induced confirmation does not allow interaction with CD94/NKG2A [23].

It remains unknown whether HLA-E can bind to additional receptors next to CD94/NKG2A/C, LILRB1/2 and the TCR. To identify these, we performed a genome-wide CRISPR/Cas9 activation screen and report two novel receptors that interact with HLA-E, the scavenger receptors Stabilin-1 (STAB1) and Stabilin-2 (STAB2). We find that these Stabilin receptors can bind HLA-E in complex with low binding affinity Mtb-derived peptides, but not when in complex with high-affinity VL9-like peptides. Furthermore, disulfide linking of the HLA-E α1 and α2 helix to promote complex stability negates the necessity for a peptide, suggesting that the conformation of the HLA-E/peptide complex, dictated by peptide affinity, plays an important role in the interaction with STAB1 or 2 [22]. Functionally, overexpression of STAB1 or STAB2 on THP-1 monocytes increased their phagocytic activity of HLA-E coated beads, suggesting this can be a physiologically relevant interaction.

## Results

### CRISPR activation screen identifies STAB1 and STAB2 as novel interactors for HLA-E

**Fig 1:**
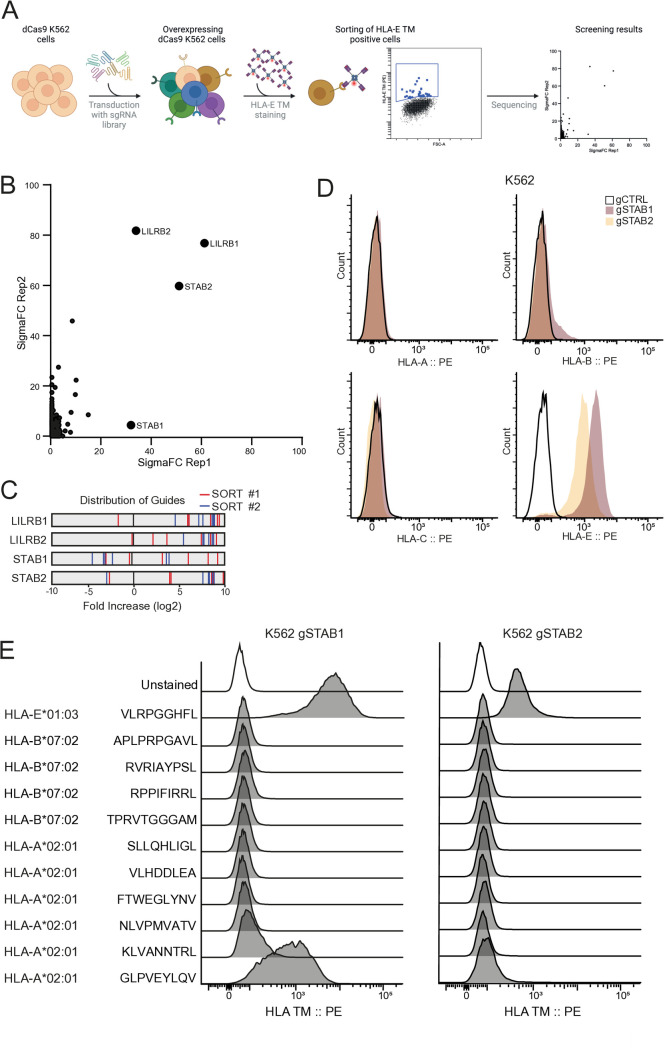
CRISPR activation screen identifies STAB1 and STAB2 as novel interactors for HLA-E. (A) Schematic overview of the receptor-ligand CRISPR activation screen. K562 cells stably expressing dCas9 were transduced with a genome-wide activation library and stained with a pool of HLA-E*01:03 tetramers loaded with 4 different peptides (VLRPGGHFL, RMPPLGHEL, VMAPRTLIL, RLPAKAPLL). Enrichment of gRNAs in sorted cell populations were determined using next generation sequencing. (B) Gene ranking scores of genes in two replicate screens were calculated using SigmaFC scores from PinAPL-Py and plotted against each other. Hits of interest are annotated. (C) Enrichment of single gRNAs for top hits of the screen, red and blue stripes represent enrichment of individual gRNAs of two replicate sorts compared to unsorted cells. (D) K562 dCas9 cells were transduced with a control guide or a gRNA upregulating either STAB1 or STAB2, stained with HLA-A, -B, -C or -E tetramers (HLA-A*02:01 NLVPMVATV, HLA-B*07:02 TPRVTGGGAM, HLA-C*07:01-VRIGHLYIL, HLA-E*01:03 RMPPLGHEL) and analysed by flow cytometry. (E) K562 gSTAB1 or K562 gSTAB2 cells were stained with HLA tetramers (HLA-A*02:01; HLA-B*07:02; HLA-E*01:03) loaded with indicated peptides and analysed by flow cytometry. All graphs represent at least two biological replicates.

To identify HLA-E binding partners in an unbiased manner, we performed a genome-wide CRISPR activation screen on K562 cells and stained potential interactors with HLA-E tetramers (Fig 1A). K562 cells stably expressing a catalytic dead Cas9 (dCas9) were transduced with a CRISPR/Cas9 activation library, resulting in a group of cells where each cell stably overexpresses one gene. A pool of HLA-E*01:03 tetramers, loaded with four different peptides with different HLA-E binding affinities (Mtb-derived peptides 44, 62 and 68, and VL9_2) was used to sort out cells based on HLA-E tetramer staining. An overview of peptides used in HLA-E tetramers in this study can be found in Table 1. Peptides used in this screen were selected based on known variable peptide binding affinity to HLA-E [7,24]. Two biological repeats decidedly confirmed *LILRB1* and *LILRB2* as hits and identified *STAB2* as a novel hit. *STAB1* was identified in one screen as a novel hit (Fig 1B and 1C). LILRB1 and LILRB2 were identified previously as receptors for HLA-E using this screen [14]. Although both LILRB1 and LILRB2 are known interactors of classical MHC-I molecules via the β_2_M subunit [15,25], STAB1 or STAB2 have never been described as HLA-E or classical MHC-I interactors. STAB1 and STAB2 are Stabilin receptors, a class of large scavenger receptors that mediate clearance of degradation products from the circulation [26–28]. Whereas the functionality of STAB1 and STAB2 overlaps in this regard, STAB1 is also involved in lymphocyte transmigration and STAB2 is almost solely responsible for the clearance of hyaluronic acid in the liver [29–31].

**Table 1:** Names, sequences and origin of peptides loaded in HLA-E in this study.

Peptide name	Peptide sequence	Origin	Affinity for HLA-E*01:03 (s/p ratio) [7]
**MTBHLAE_30**	VLPKRARLL	Mtb	0,43
**MTBHLAE_31**	VLPAKLILM	Mtb	0,20
**MTBHLAE_34**	LLPIKIPLI	Mtb	0,20
**MTBHLAE_62**	ALQSAAPWL	Mtb	0,59
**MTBHLAE_63**	ILAFEAPEL	Mtb	0,32
**MTBHLAE_65**	ILLSRVPEL	Mtb	1,00
**MTBHLAE_81**	VLPAAPWL	Mtb	0,62
**MTBHLAE_87**	KLSTLTPYL	Mtb	0,70
**MTBHLAE_93**	RLEAVVMLL	Mtb	0,29
**p34**	VMTTVLATL	Mtb	0,22
**p44**	RLPAKAPLL	Mtb	1,24
**p55**	VMATRRNVL	Mtb	0,02
**p62**	RMPPLGHEL	Mtb	0,09
**p68**	VLRPGGHFL	Mtb	0,11
**VLA**	VLAPRTLLL	CMV	1,00
**VL9_1**	VMAPRTLLL	MHC-I	1,00
**VL9_2**	VMAPRTLIL	MHC-I	1,00

Mtb, *Mycobacterium Tuberculosis*; CMV, *cytomegalovirus*; MHC, major histocompatibility complex.

To validate the screening results, we generated K562 cells overexpressing STAB1 or STAB2 using guide RNA (gRNA)-mediated activation and stained them with HLA-A, -B or -C tetramers (loaded with NLVPMVATV, TPRVTGGGAM or VRIGHLYIL peptides, respectively) or with the HLA-E/p62 tetramer used in the screen. Notably, none of the classical HLA-I tetramers stained STAB1 or STAB2 overexpressing cells while HLA-E/p62 tetramers stained both (Fig 1D). To substantiate specificity for HLA-E, we tested ten additional HLA-A and -B tetramers, including the HLA*02:01 allotype, which is very similar to HLA-E in terms of peptide binding repertoire [32]. None of the tested HLA-A or -B tetramers showed binding to STAB1 or STAB2 cells, except the HLA-A*02:01 tetramer loaded with Mtb peptide GLPVEYLQV, though the measured signal was low compared to HLA-E/p62 tetramer staining and only binding to STAB1 was observed (Fig 1E). Thus, using a CRISPR activation screen we identified STAB1 and STAB2 as two novel binding partners that seem to interact with HLA-E/peptide complexes.

### STAB1 or STAB2 overexpressing cells interact with HLA-E presenting low affinity Mtb peptides, but not high-affinity HLA-I leader-sequence derived peptides

**Fig 2:**
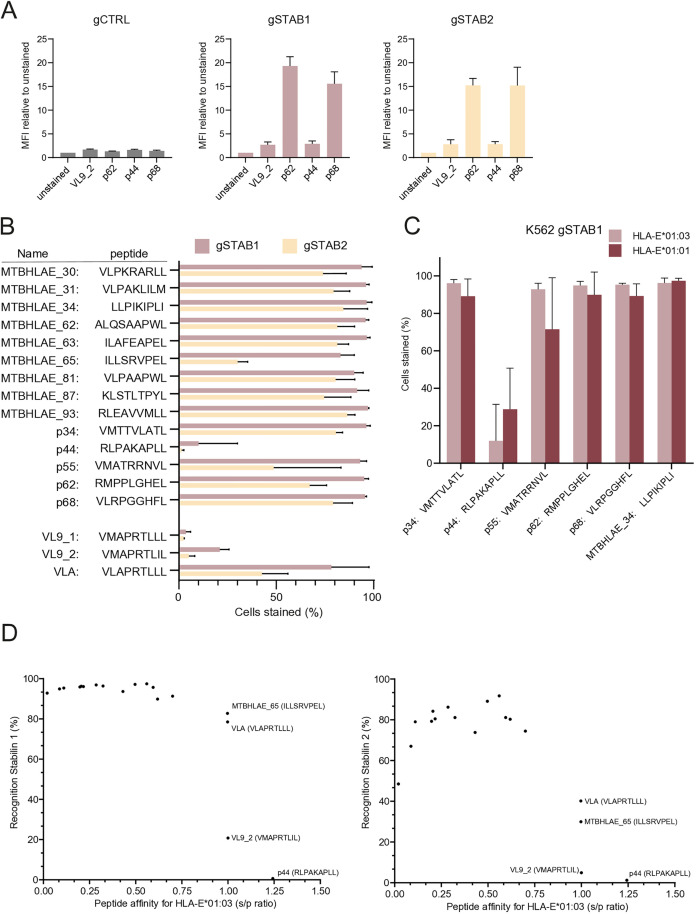
STAB1 or STAB2 overexpressing cells interact with HLA-E presenting low affinity Mtb peptides, but not high-affinity HLA-I leader-sequence derived peptides (A) K562 dCas9 cells transduced with either a control guide or a guide targeting STAB1 or STAB2 were stained with HLA-E*01:03 tetramers used in the screen and analysed by flow cytometry. Data are represented as mean ± SD. (B) K562 gSTAB1 or K562 gSTAB2 cells were stained with HLA-E*01:03 tetramers loaded with different peptides and analysed by flow cytometry. The percentage of cells with signal above unstained controls is plotted as mean ± SD. (C) K562 gSTAB1 cells were stained with peptide loaded HLA-E*01:01 or HLA-E*01:03 tetramers and analysed by flow cytometry. (D) Correlation of peptide affinity for HLA-E with the tetramer staining on K562 gSTAB1 or gSTAB2 cells. Peptide affinity is represented as a signal to positive ratio (S/P ratio), as described previously based on the absorbance at 450 nm [33]. The peptides were normalized to the high-affinity peptides VMAPRTLIL and VLAPRTLL. Hence, these peptides have per definition a S/P ratio of 1. Data are represented as mean ± SD. All data represent at least three independent experiments replicates.

In the activation screen we used a pool of HLA-E tetramers folded with four different peptides with different binding affinities to increase detection of potential binders. Staining STAB1 or STAB2 overexpressing K562 cells with the individual tetramers showed that STAB1 or STAB2 interacted with HLA-E*01:03 tetramers loaded with p62 and p68, while minimal staining was observed for HLA-E*01:03 tetramers loaded with VL9_2 and p44 (Fig 2A). To assess the peptide specificity of these interactions in more detail we expanded the repertoire of tetramers and included 11 additional Mtb peptides with varying affinities for HLA-E, a second VL9 peptide (VL9_1) and the UL40-derived VL9 mimic from CMV (VLA) (Table 1). All tetramers loaded with Mtb peptides could bind STAB1 or STAB2 overexpressing cells, except for p44 where little binding was observed (Fig 2B). This observation is consistent with the fact that p44 has higher affinity for HLA-E than all other Mtb peptides evaluated [18] and induces a distinct conformation in the alpha chain of HLA-E similar to VL9 peptides [22]. Therefore, the conformation of the HLA-E/peptide complex is likely to play a role in the interaction with STAB1 or 2.

Although HLA-E*01:01 and 01:03 allotype do not seem to affect binding characteristics to the other receptors of HLA-E, the binding site of STAB1 or STAB2 to HLA-E remains unknown. We observed no significant difference in staining between the two HLA-E allotypes, since five HLA-E/Mtb tetramers stained K562 gSTAB1 cells irrespective of allotype, and both HLA-E*01:01 and 01:03 tetramers loaded with p44 showed little binding (Fig 2C). Correlation of Stabilin binding to HLA-E tetramers with the affinity of the loaded peptide shows that the interaction is dependent on the affinity of the loaded peptide (Fig 2D), with high affinity peptides rendering HLA-E unable to bind STABs. For STAB2, very low affinity complexes also failed to bind, possibly because of complex dissociation. Collectively, these results demonstrate that the Stabilin-HLA-E interaction is independent of HLA-E allotype, and is observed for a series of Mtb peptides, but not for leader sequences from HLA class Ia alleles. This suggests that Stabilins discriminate between HLA-E loaded with high- and low-affinity peptides which form distinct HLA-E/peptide conformations.

### Conformation of the HLA-E/peptide complex is likely involved in the interaction with STAB1 or STAB2

**Fig 3:**
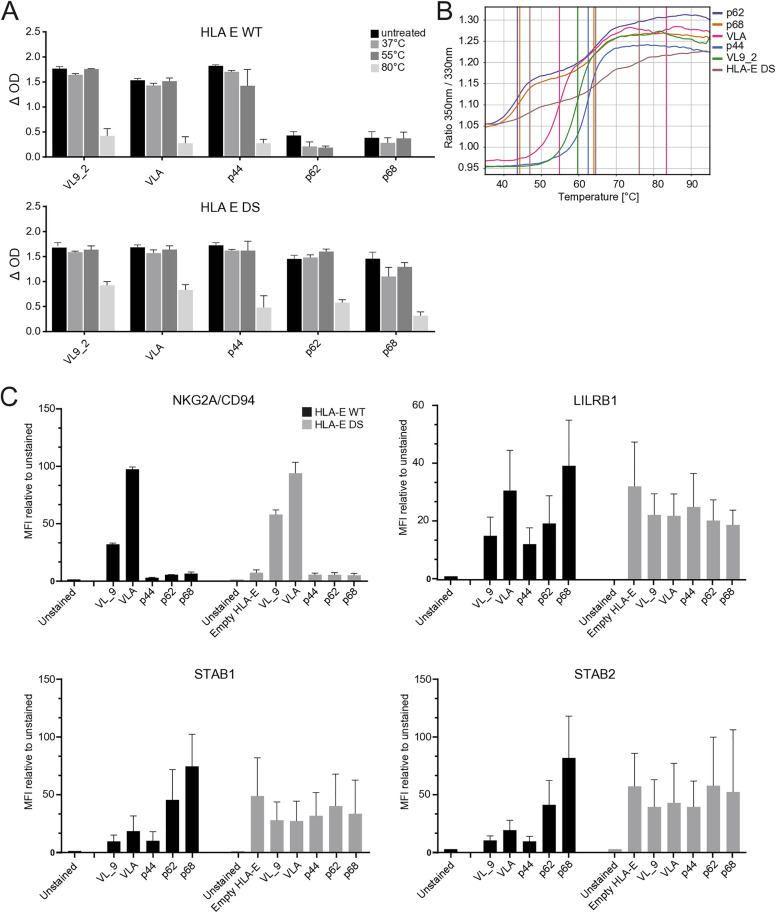
Conformation of the HLA-E/peptide complex is likely involved in the interaction with STAB1 or STAB2 (A) HLA-E monomers in complex with indicated peptides were exposed to increasing temperatures to measure their stability. Binding of stable complexes was assessed by sandwich ELISA. (B) Melting curves of HLA-E/peptide complexes. (C) HLA-E monomers stabilized by a disulfide bond (gray) were folded with indicated peptides or without peptide. WT HLA-E tetramers (black) were folded with indicated peptides. After tetramerization, binding to K562 cells overexpressing CD94/NKG2A or K562 dCas9 cells transduced with a guide targeting LILRB1, STAB1 or STAB2 was assessed by flow cytometry. Data are represented as mean ± SD. All data represent at least two independent replicates.

The affinity of Mtb-derived peptides for HLA-E is low, and a previous study showed that HLA-E can be stable without peptide [22], therefore empty or unfolded HLA-E complexes could have contributed to the binding of STAB1 and 2 to HLA-E/peptide complexes. To assess potential unfolding, we tested the stability of our HLA-E/peptide complexes using a thermostability assay. Specifically, we examined the stability of HLA-E in complex with lower-affinity peptides by exposing HLA-E monomers folded with p44, p62, p68, VLA and VL9_2 to increasing temperatures and measured complex persistence in a sandwich ELISA [24]. Only exposure to 80^o^C markedly reduced the stability of the complex, whereas HLA-E monomers exposed to 37^o^C and 55^o^C were nearly as stable as unheated monomers (Fig 3A). Consistently, melting temperature analysis revealed that HLA-E/p62 and 68 monomers were stable up to 45^o^C, whereas the other HLA-E/peptide monomers were stable up to 60^o^C (Fig 3B). The stability of these HLA-E/peptide complexes at higher temperatures strongly suggests that unfolded HLA-E monomers did not contribute to the staining results for STAB1 or STAB2.

Next, we tried to dissect whether empty or low-affinity peptide loaded HLA-E governed the interaction with STAB1/2. Because we were unable to produce stable, empty HLA-E complexes that passed quality control, we generated an empty HLA-E mimic via introduction of a disulfide (DS) bond between the α1 and α2 helix of HLA-E, referred to as HLA-E DS [34,35]. This bond stabilizes the complex and allows the production of empty HLA molecules. HLA-E DS monomers were folded with p44, p62, p68, VLA, VL9_2, yielding stable complexes at similar levels as high-affinity complexes without DS (Fig 3A). Binding was tested by staining of K562 cells overexpressing CD94/NKG2A, demonstrating that only HLA-E DS loaded with VL_9 or VLA interacted, as anticipated (Fig 3C). Furthermore, all complexes interacted with LILRB1 expressing cells, showcasing proper folding and assembly. Staining of STAB1 and STAB2 cells with HLA-E DS tetramers revealed a strong binding capacity regardless of the folded peptide, whereas staining with conventional HLA-E tetramers was again peptide dependent, preferring lower-affinity peptides. Even HLA-E DS formed without peptide was able to bind STAB1/2 cells. Since the introduction of a DS bond affects the conformation of the HLA-E complex, this suggests that a specific HLA-E conformation may be crucial for the interaction with STAB1 or STAB2. The interaction is thus unlikely to be directly dependent on the presented peptide itself, but rather on the conformational change in HLA-E induced by certain peptides. Together, these findings demonstrate that the tested HLA-E/peptide complexes are stable and that the conformation of the HLA-E/peptide complex, as induced by some peptides, likely plays a crucial role in the interaction with STAB1 or STAB2.

### STAB1 or STAB2 expression increases THP-1 phagocytotic uptake of HLA-E coated beads

**Fig 4:**
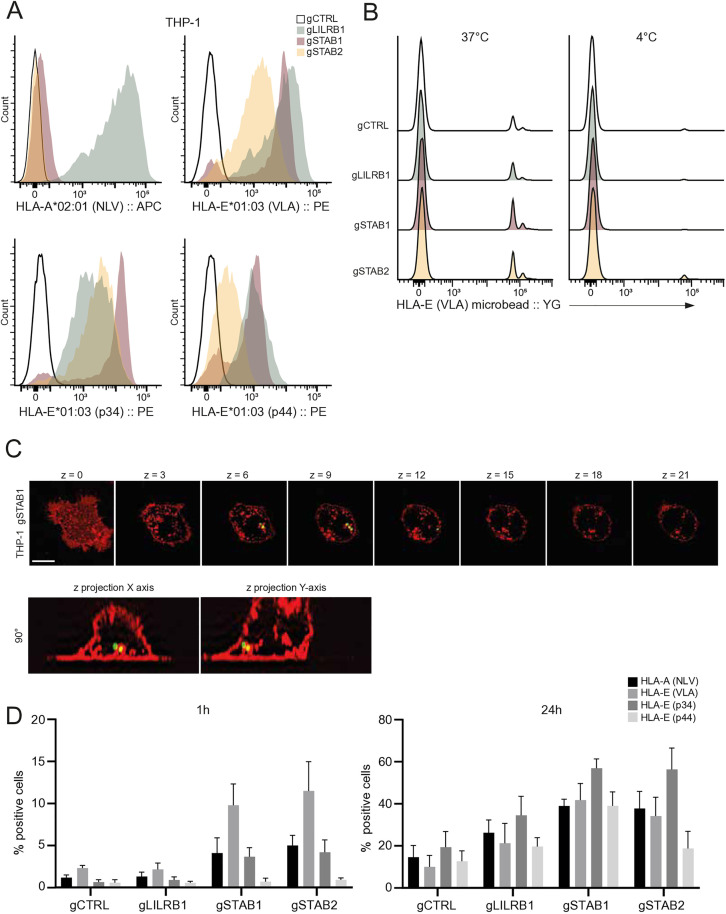
STAB1 or STAB2 expression increases THP-1 phagocytotic uptake of HLA-E coated beads (A) THP-1 dCas9 cells were transduced with a control guide or a guide targeting either LILRB1, STAB1 or STAB2 stained with HLA tetramers and analysed by flow cytometry. (B) THP-1 cells were incubated with HLA-E (VLA) coated microbeads at either 37°C or 4°C and analysed by flow cytometry. (C) THP-1 cells were incubated with fluorescent microbeads coated with peptide-loaded HLA molecules for either 1h or 24h and analysed using flow cytometry. The percentage of cells with signal above untreated controls is plotted as mean ± SD. (D) Representative Z-stack of images gathered by confocal microscopy of a THP-1 gSTAB1 cell incubated with HLA-E (VLA) coated fluorescent beads (green). CellMask membrane staining is depicted in red. All data represent at least three independent experiments.

STAB1 and STAB2 are known to bind various ligands to induce cell-cell interactions or phagocytotic uptake of extracellular ligands. Since the THP-1 monocytic cell line is capable of phagocytosis [36], we generated THP-1 cells overexpressing either LILRB1, STAB1 or STAB2 to test the scavenger function of STAB1 and STAB2 in the context of HLA-E. Generated cell lines were stained with HLA tetramers to validate HLA binding capacity. THP-1 gLILRB1 cells could bind all (classical as well as nonclassical HLA I) tetramers, whereas THP-1 gSTAB1 or gSTAB2 cells could only bind HLA-E*01:03 VLA, p34 or p44 tetramers and not HLA-A*02:01 NLVPMVATV tetramers (Fig 4A). To assess the interaction in a functional manner, THP-1 cells were incubated with fluorescent microspheres coated with HLA molecules and bead uptake was measured by flow cytometry. All THP-1 cell lines could take up HLA-E/VLA coated beads at 37°C while little to no uptake was observed when cells were incubated at 4°C (Fig 4B), suggesting the measured signal originated from actively phagocytosed beads. To confirm this, we analysed the uptake and intracellular localization of beads using microscopy, where we generated a Z-projection of THP-1 gSTAB1 cells after incubation with HLA-E/VLA beads. Signal originated from a sphere that localized above the lower membrane staining and below the upper membrane, implying the bead is located intracellularly (Fig 4C). Analysis of the different cell lines showed that after 1h of incubation, gSTAB1 and gSTAB2 THP-1 cells displayed increased uptake of all beads except p44 loaded HLA-E compared to gCTRL or gLILRB1 cells (Fig 4D). Bead uptake was highest for HLA-E/VLA coated beads and no uptake was observed for HLA-E/p44 beads in any cell line. Interestingly, after 24h the increase in bead uptake of STAB1 and STAB2 cells persisted and uptake of HLA-E/p34 beads was highest for all cell lines compared to other beads. Thus, both STAB1 and STAB2 overexpressing THP-1 cells have increased phagocytic capacity of HLA-E coated beads compared to LILRB1 overexpressing THP-1 cells, suggesting that STAB1 and STAB2 interact with HLA-E in a functional manner.

## Discussion

In this study, we used a CRISPR/Cas9 activation screen to identify STAB1 and STAB2 as novel interactors for HLA-E. We characterized this interaction using different HLA tetramers and found that while most HLA-A and -B tetramers did not bind to STAB1 or STAB2, HLA-E tetramers loaded with low-affinity Mtb peptides could interact. Stabilin/HLA-E interactions thus differentiated between lower affinity HLA-E peptides, which were derived from Mtb, and higher affinity HLA-E peptides including leader sequence self-peptides, which did not permit HLA-E/Stabilin interactions. These results suggest STAB1 and STAB2 can engage in interactions with HLA-E and discriminate between peptides or peptide associated conformations of HLA-E.

Structurally, STAB1 and STAB2 are composed of seven fasciclin domains and four EGF-like clusters and are 55% homologous in their extracellular domains [37]. The staining pattern of STAB1 or STAB2 overexpressing cells with HLA-E tetramers was comparable, suggesting the interaction site is located at one of the homologous domains of STAB1 and STAB2. In general, cells expressing STAB1 stained more intensely with HLA-E tetramers, arguing that either its expression was higher, or that STAB1 has slightly higher affinity for HLA-E than STAB2. In line with this, STAB1-expressing cells had some binding of HLA-E/p44 complexes, especially in THP-1 cells. So, while the affinity of STAB for low affinity peptide loaded HLA-E is clearly higher, there is some binding to other HLA-E complexes as well.

Although our initial experiments revealed no detectable difference between HLA-E*01:01 and HLA-E*01:03 in binding to STAB1 or STAB2, there may be allele-specific differences in peptide affinity, stability, and functional outcomes across various disease contexts. For instance, it has been suggested that the HLA-E*01:03 allele occurs more frequently compared to HLA-E*01:01 in individuals with SARS-CoV-2 infection than in uninfected individuals [38]. However, it is still unclear how differences in this allele influence the induction and function of the T cell response during infection. Additionally, using an ELISA-based peptide affinity assays we showed that Mtb-derived peptides can vary in affinity for HLA-E*01:01 and HLA-E*01:03 [7]*.* Such distinctions between alleles should be carefully considered in future studies that involve physiological or disease-relevant models.

The interaction of HLA-E with STAB1 or STAB2 was peptide dependent, however the physiochemical diversity of the Mtb peptide repertoire tested renders it unlikely that the peptide directly interacts with STAB1 or STAB2. Instead, distinct structural HLA-E conformations seem more likely to explain the observed variation, since high-affinity peptides mediate a conformational change in the HLA-E alpha2 helix which generates a distinct molecular antigenic surface compared to low-affinity non-VL9 peptides and empty HLA-E [22]. These findings are in accord with another study that used a disulfide bridge to stabilize HLA molecules, as they observed that the PBG of empty HLA-A*02:01 DS molecules has a different conformational state compared to the peptide-bound form, revealing that the antigenic surface of empty HLA-I molecules is different compared to peptide-bound molecules [39].

We observed that DS-stabilized HLA-E interacted with CD94/NKG2A in a peptide-dependent manner, indicating that the HLA-E DS retained regular peptide binding dynamics. Interestingly, STAB1/2 bound to HLA-E DS independently of the presented peptide and even in absence of peptide. This suggests that the disulfide bridge forces HLA-E in a specific conformation that can bind to STAB1/2, possibly resembling the conformation of HLA-E molecules bound to low-affinity peptides. This hypothesis argues for the existence of a general structural variant of HLA-E, likely induced by low-affinity peptides, that facilitates STAB1/2 binding, which is abolished by binding of high-affinity peptides such as VL9. In contrast, the interaction of CD94/NKG2A with HLA-E is more focused on peptide sequences. In line with additional functions for HLA-E beyond CD94/NKG2A, we recently showed that the cell-surface half-life of mouse MHC-E (Qa-1) is much longer than that of MHC-I leader peptide loaded Qa-1, arguing for the existence of a large pool of empty cell surface MHC-E or surface peptide stabilized MHC-E [14]. Others have also demonstrated the existence of empty HLA-E sequences in vitro [21], putting forward the notion that empty HLA-E complexed with β_2_M has a degree of stability. Although we failed to produce empty HLA-E complexes, DS-stabilized HLA-E/β_2_M complexes without peptide could interact with STAB1/2. These results therefore provide a possible function for HLA-E in complex with peptides that do not induce the distinct HLA-E/VL9 conformation, or even ‘empty’ HLA-E. This discrimination would enable STAB1 and STAB2 to distinguish VL9-loaded HLA-E from other HLA-E variants. As CD94/NKG2A only recognizes VL9 bound HLA-E, this would provide HLA-E with distinct binding partners based on peptide affinity. Endogenous high-affinity peptides facilitate binding to the NKG2A receptor, while low-affinity peptides enable binding to Stabilin receptors.

STAB1 is expressed on lymphatic endothelium, where it is involved in lymphocyte adhesion and transmigration [29,30,40], however it is unknown what ligands it specifically binds to perform this function. Healthy tissue-resident macrophages with VL9-HLA-E need to stay in tissue for immune surveillance, while macrophages with pathogenic peptide-bound HLA-E require transmigration towards the lymph nodes, for example to initiate cross presentation and T cell priming to immune-control pathogens. This means it is essential that lymphatic endothelial cells can distinguish between HLA-E bound to VL9 and pathogen derived peptides. The expression of STAB1 and STAB2 on endothelium and the interaction with HLA-E bound to pathogen derived peptides, provides a compelling hypothesis where Stabilin receptors on lymphatic endothelium can discriminate between infected and healthy macrophages via HLA-E. Lymphocyte trafficking by STAB1 occurs under physiological and inflammatory conditions, therefore it is unlikely that HLA-E bound to pathogenic peptides is the sole ligand. However, this interaction would promote more specific adhesion of infected macrophages and can serve as danger sensing to the immune system. Migration assays using endothelial cell lines that express STAB1 or STAB2 and lymphocytes with specific HLA-E/peptide complexes would be crucial to determine any biological significance of this interaction in this context.

Collectively, we identified STAB1 and STAB2 as novel receptors for HLA-E, which preferentially recognize HLA-E presenting lower affinity, pathogen-derived peptides, likely reliant on the HLA-E/peptide conformation. This interaction may serve as a danger signal to the immune system. Further functional characterization of this interaction is required to understand its contribution in immune surveillance and tissue migration.

### Limitations

Our study identifies a novel interaction between Stabilin receptors and HLA-E. We tested Mtb-derived peptides for Stabilin 1 and 2 recognition, however peptides from other pathogens, should be evaluated as well to understand if our findings can be translated to other pathogens. Due to a lack of reliable commercial antibodies for STAB1 and STAB2, we used qPCR to validate our generated overexpression cell lines. Furthermore, it would be valuable to determine the molecular affinity of the Stabilin/HLA-E interactions, since *in vivo* competition may occur with other ligands of HLA-E, specifically LILRB1, CD94/NKG2A or -C and TCRs. Alternatively, competition between receptors might also be mediated by the peptide presented on HLA-E, allowing interaction with STAB1 and STAB2 when HLA-E presents pathogen-derived peptides compared to VL9 peptides. Endothelial cell lines that express STAB1 or STAB2 would be critical to perform migratory assays to measure the potential biological significance of this interaction in the context of lymphocyte migration.

## Materials and methods

### Animal ethics statement

An animal research ethics committee was not required as no animals or animal data was used in this study.

### Cell culture

HEK293T cells were maintained in DMEM supplemented with 8% fetal calf serum (FCS). K562 (ATCC, Cat #CCL-243) and THP-1 (ATCC, TIB-202) cells were cultured in RPMI supplemented with 8% FCS. K562 cells stably expressing CD94/NKG2A or over-expressing STAB1/2 or LILRB1 were cultured at 37^o^C in Roswell Park Memorial Institute (RPMI) 1640 medium (Gibco) + 10% Fetal Bovine Serum (FBS) (Capricorn) + 1% Penicillin/Streptomycin (P/S) (Gibco). All cell lines were regularly checked for the absence of mycoplasma and authenticated using STR profiling.

### Transfections transductions

Transfections were performed using polyethyleneimine (Polysciences). For viral particle generation, HEK293T cells were transfected with the lentiviral construct of interest and three packaging plasmids: pCMV-VSV-G (Addgene #8454), pMDLg/pRRE (Addgene #12251) and pRSV-Rev (Addgene #12253). Supernatant containing viral particles was harvested 48h after transduction, filtered and used to transduce target cells with 8 µg/ml polybrene (EMD Millipore).

### Generation of and staining with conventional and disulfide-linked HLA-E tetramers

Tetramers were generated in-house as described previously [41,42]. HLA-E DS tetramers were generated by introducing a Y84C and A139C mutation to generate a disulfide bridge for stabilization [39]. HLA-E DS tetramers were folded with a dipeptide, after which they were loaded with a peptide and tetramerized. All peptides used in this study were obtained from Peptide2.0 Inc. with >99% purity. Tetramer staining on K562 STAB1/2, LILRB1, CD94/NKG2A or wildtype cells was performed for 20 min at 37°C in PBS supplemented with 0.1% bovine serum albumin (BSA) at a 10nM concentration in the dark. Cells were washed in PBS 0.1% BSA for 7 min at 1400 rpm, fixated in 1% paraformaldehyde and washed again in PBS 0.1% BSA for 7 min at 1400 rpm. Cells were acquired on a BD LSR Fortessa (BD Bioscience) and analyzed in FlowJo software v10.8.1.

### HLA-E/peptide monomer stability measurements via ELISA and thermal melting assays

HLA-E DS or conventional monomers folded or unfolded (only for DS) with peptides were produced in-house as described in detail previously [41]. HLA-E monomers were exposed to 37^o^C, 55^o^C and 80^o^C for 10 minutes in a heat-block or were left at RT. 25 µL of 0.001 µg/µL heat-exposed or non-exposed monomer was added to anti-human HLA-E monoclonal antibody (clone 3D12, BioLegend) coated 96-well half area ELISA microplates (Greiner-Bio). HLA-E monomer integrity was evaluated by addition of horseradish peroxidase (HRP)-conjugated anti-β2m antibodies (Thermo Fisher) and the signal was amplified using HRP-coupled goat anti-rabbit IgG antibodies (Dako) [41]. Absorbance was measured at 450 nm using a SpectraMax i3x reader (Bio-Rad) and absorbance is positively related to the stability of the HLA-E monomers. Thermal melting assays were performed with a Tycho (NanoTemper).

### Genome-wide CRISPR activation screening for HLA-E tetramer binding

CRISPR activation screens were performed by transducing K562 cells with dCas9-Blast (Addgene #61425), a kind gift from Feng Zhang, and the Calabrese genome-wide activation library (sublibrary A + B), kindly provided by John Doench (Addgene # 1000000111). Transduced cells were selected using blasticidin (5 µg/ml) and puromycin (2 µg/ml). After seven days two sets of 30 million cells were stained with a pool of PE-labeled HLA-E tetramers (10nM) loaded with a VMAPRTLIL, RMPLLGHEL, RLPAKAPPL or VLRPGGHFL peptide. Cells that stained positive (between 0.05 and 0.5%) were sorted using an Aria cell sorter and genomic DNA (gDNA) was isolated from the sorted and unsorted populations using an isolate II genomic DNA kit (GC Biotech). gDNA was amplified using the established protocol [43]. Inserts were mapped to the reference and gRNA enrichment was analyzed using PinAPL-Py [44].

### Hit validation

To validate hits from the overexpression screens, individual gRNAs were cloned into the pXPR_502 vector (Addgene #96923) and K562 cells stably expressing dCas9 were transduced and selected using puromycin (2 µg/ml). Guide sequences for specific genes are: LILRB1: 5’- AAGACTCAGAGATTTGTTCC −3’, STAB1: 5’- GCTGGCCACTGACCCCCCAT −3’, STAB2: 5’- GAGGGGGCAGAGTAGAGGAG −3’. A non-targeting guide from the calabrese library was used in validation experiments as a control: 5’- AAAAAGCTTCCGCCTGATGG-3’.

### Bead uptake assay

Beads used in the assay were generated by coupling 10 µl yellow-green fluorescent neutravidin-labeled microspheres (Thermo Fisher, #8776) to 6,75 µg biotinylated recombinant HLA monomers (generated in-house). After 2 hrs of incubation at RT beads were washed several times with PBS and resuspended in 190 µl PBS.THP-1 cells stably expressing dCas9 were transduced with the pXPR_502 vector containing either a control guide or a guide targeting LILRB1, STAB1 or STAB2 using the sequences mentioned earlier. 20.000 cells were incubated with 150.000 beads at 37°C in a total volume of 50 µL in a 96-wells plate. After washing, cells were analysed by flow cytometry as described below.

### Flow cytometry

Flow cytometry was conducted on an LSR-II, LSRFortessa flow cytometer (BD Biosciences) or Aurora 3L (Cytek Biosciences) and data was analyzed using FlowJo (V10.8). Cell sorting was performed on a BD FACSARIA III (BD Biosciences).

### Microscopy

After bead uptake, THP-1 cells were stained with CellMask Deep Red (Thermo Fisher, C10046) for 15 min at room temperature. After washing, cells were seeded in 4-chamber live cell dishes and imaged on a Leica SP8 with an Andor Dragonfly spinning disc module. 0,2µm Z stacks were acquired with a 63x oil immersion objective and processed using ImageJ or Imaris software for 3D rendering.
